# Emerging therapies in the management of macular edema: a review

**DOI:** 10.12688/f1000research.19198.1

**Published:** 2019-08-12

**Authors:** Riccardo Sacconi, Chiara Giuffrè, Eleonora Corbelli, Enrico Borrelli, Giuseppe Querques, Francesco Bandello

**Affiliations:** 1 Department of Ophthalmology, University Vita-Salute, IRCCS Ospedale San Raffaele, Via Olgettina 60, Milan, 20132, Italy

**Keywords:** corticosteroid, diabetic macular edema, macular edema, retinal vein occlusion, uveitic macular edema, uveitis, vascular endothelial growth factor

## Abstract

Macular edema (ME) is a major complication of several vascular and inflammatory retinal diseases. Multiple mechanisms are implicated in its development and lead to visual impairment that could be reversible (the acute stages) or not reversible (long-standing ME). For this reason, an effective approach to the treatment of ME is of paramount importance in order to prevent irreversible damage of visual function. In this review, we discuss the management of ME and, in particular, current data of studies and clinical trials about drugs that have already been evaluated or are under investigation in the management of ME. Although several diseases could lead to the development of ME, we focus on the three main causes: diabetic retinopathy (DR), retinal vein occlusion (RVO), and uveitis. The introduction into clinical practice of anti-vascular endothelial growth factor injections (ranibizumab and aflibercept) and dexamethasone implants has revolutionized the treatment of ME secondary to DR and RVO. However, new drugs are needed in the treatment of resistant forms of ME secondary to DR and RVO. A fluocinolone acetonide implant has been approved by the US Food and Drug Administration for the treatment of diabetic ME but not for RVO. Furthermore, brolucizumab and abicipar pegol have been shown to be effective in preliminary studies and have the chance to be approved soon for diabetic ME treatment. In ME secondary to uveitis, a crucial role is played by corticosteroids and non-biologic immunomodulatory drugs. However, several new biologic agents are under investigation in different clinical trials and could be important new therapeutic options in cases with a low response to first-line therapy. However, only a few of these drugs will enter the market after proving their safety and efficacy. Only after that will we be able to offer a new therapeutic option to patients affected by uveitic ME.

## Introduction

Macular edema (ME) is the major cause of visual impairment in several metabolic, vascular, and inflammatory retinal diseases. It affects about 7 million patients with diabetic retinopathy (DR) ^1^ and 3 million patients with retinal vein occlusion (RVO) ^2^ and causes a visual decline in 40% of patients affected by uveitis ^3^. ME is characterized by an abnormal presence of fluid inside the retinal layers of the macula ^4^. Normally, there is a fine balance between fluid that enters and exits inside the retinal layers. This balance is necessary for the retinal homeostasis in order to obtain the tissue transparency. However, for several diseases, this balance could be compromised, resulting in an imbalance between fluid that enters and exits and thus causing ME development. The accumulation of the fluid could be extracellular (resulting in cystic space formation), intracellular (causing a swelling of the cells), or subretinal (causing the accumulation in the subretinal space) ^4– 6^. The regulation of fluid and molecules that enter and exit from the vitreous, retinal vessels, and choroidal vessels is controlled by structures forming the inner and the outer blood-retinal barriers ^7, 8^. The inner blood-retinal barrier is composed of the tight junctions between the endothelial cells of the capillaries inside the retinal plexuses, astrocytes, Müller cells, pericytes, and smooth muscle cells ^9– 11^. On the other hand, the outer blood-retinal barrier is composed of the retinal pigment epithelium (RPE) cells and their inter-cellular junctions that regulate the passage of fluid and molecules from the choroidal vessels to the neurosensory retina ^12^.

ME causes visual impairment that could be reversible or permanent. Multiple mechanisms are implicated in the development of visual impairment due to ME. During the acute phase of ME development, the imbalance of fluid that enters and exits inside the retinal layers causes a retinal hydration state that interferes with the passage of light through the neuroretinal layers. These alterations lead to an acute effect on visual function, such as central vision loss, a relative central scotoma, metamorphopsia, impaired stereopsis, or disturbed color vision ^13, 14^. In this phase, the treatment or the spontaneous resolution of ME is able to reverse the visual impairment. On the other hand, long-standing ME causes irreversible changes of the retinal layers, leading to a permanent deficit in visual function. The main anatomical damage caused by chronic ME is linked to alterations of the outer limiting membrane, photoreceptor segments (outer nuclear layer thinning and outer segment atrophy), and disorganization of inner retinal layers ^15– 17^. For these reasons, the treatment of ME should be performed as soon as possible in order to avoid structural retinal changes and irreversible damage of visual function.

In this review, we focus on the management of ME and, in particular, on current data of studies and clinical trials about drugs that are under investigation or have already been evaluated in ME management. Although several diseases could lead to the development of ME (that is, DR ^1^, RVO ^2^, uveitis ^3^, Irvine–Gass postoperative ME ^18^, idiopathic macular telangiectasia type 1 and 2 ^19, 20^, perifoveal exudative vascular anomalous complex ^21^, paclitaxel-induced maculopathy ^22^, MEK inhibitor–associated maculopathy ^23^, hypoproteinemia ^24^, paracentral acute middle maculopathy ^25^, and central serous chorioretinopathy ^26^), we will discuss the management of the major causes of ME, including DR, RVO, and uveitis.

## Management of macular edema: diabetic retinopathy

Diabetic macular edema (DME) is the first cause of visual loss in patients with diabetes ^27^. For several years, grid and focal laser photocoagulation were considered the standard of care for DME. However, thanks to the introduction into clinical practice of intravitreal anti-vascular endothelial growth factor (anti-VEGF) injections and corticosteroid implants, the treatment of DME has been completely revolutionized. Anti-VEGF molecules, such as ranibizumab (Lucentis, Novartis Europharm Ltd, Horsham, UK) and aflibercept (Eylea ^®^, Bayer Pharma, Berlin, Germany), demonstrated a benefit ratio and superior efficacy compared with the previous standard of care (laser photocoagulation) in large phase 3 clinical trials ^28– 32^. The encouraging results in terms of clinically relevant improvement of visual acuity, reduction of fluid accumulation, and decreased severity of DR led to their approval for DME treatment by the US Food and Drug Administration (FDA) starting in 2012. Owing to the efficacy and safety profile of anti-VEGF therapy, it has become the first-line treatment. Besides ranibizumab and aflibercept, bevacizumab demonstrated efficacy in the treatment of DME. Bevacizumab is a monoclonal antibody used to treat a number of types of cancer, although its intraocular use is considered off-label. The Diabetic Retinopathy Clinical Research Network (DRCR.net) evaluated the outcomes of patients with DME treated with bevacizumab, ranibizumab, or aflibercept and reported best outcomes in patients whose DME was treated with aflibercept with visual acuity of 20/50 or worse at baseline ^33^.

Ziv-aflibercept (Zaltrap; Sanofi-Aventis, Bridgewater, NJ, USA/Regeneron Pharmaceuticals, Tarrytown, NY, USA) is a fusion protein approved by the FDA for the treatment of metastatic colorectal carcinoma. Although aflibercept and ziv-aflibercept are structurally identical, they differ for the buffer. This difference results in a higher osmolarity of ziv-aflibercept compared to aflibercept. De Andrade *et al*. ^34^ tested the safety and efficacy of ziv-aflibercept in seven eyes affected by DME and disclosed no systemic or ocular complications during a 48-week follow-up. Furthermore, Mansour *et al*. ^35^ reported the off-label intraocular use of ziv-aflibercept in the treatment of 107 eyes affected by retinal diseases and concluded that ziv-aflibercept appeared safe and efficacious. However, further randomized studies are needed to demonstrate the safety and efficacy of intraocular use of ziv-aflibercept.

Corticosteroid implants are usually used as a second-line treatment for patients without significant response to anti-VEGF injections. In 2014, an intravitreal implant with 700 μg of sustained-release biodegradable dexamethasone (Ozurdex ^®^, Allergan, Inc., Irvine, CA, USA) was approved by the FDA for the treatment of DME. The MEAD study demonstrated the efficacy of dexamethasone implant in the treatment of DME with an acceptable safety profile and a low number of implants (four or five injections over a 3-year follow-up) ^36^.

Despite the strong efficacy achieved with anti-VEGF and corticosteroid therapies, a significant proportion of patients do not experience clinically meaningful improvements in vision in the real world with persistent or resistant forms of DME ^37– 39^. Moreover, frequent intravitreal administration is required to achieve and, in some cases, to maintain the observed early benefits of DME treatment over a long period of time, imposing a significant burden on patients, caregivers, treating physicians, and the health-care system. Both of these factors formed the impetus for developing new drugs and alternative methods of administration for the treatment of DME.

Novel treatment strategies for DME could be classified into four subgroups: a new slow-release sustained-delivery system for intraocular steroid, next-generation anti-VEGF-A drugs, combination drugs, and suprachoroidal corticosteroids.

### New slow-release sustained-delivery system for intraocular steroid

***Iluvien.*** Iluvien (Alimera Sciences, Alpharetta, GA, USA) is an injectable non-biodegradable intravitreal insert for sustained release of fluocinolone acetonide (FAc), a potent glucocorticoid receptor agonist, for up to 36 months. The implant, inserted into the vitreous cavity via a 25-gauge needle, contains 0.19 mg of FAc and provides a release rate of 0.2 μg/day. Iluvien is usually used for the treatment of DME in patients who previously received a course of corticosteroids and did not have a significant increase in eye pressure. The clinical efficacy of Iluvien has been evaluated in a phase 3 FAME clinical trial and confirmed by several real-life reports ^40– 42^. Of note, the continuous dosing ensures the treatment even in the case of delay to follow-up appointments. This is a fundamental advantage given that many factors can change the intensive dosing schedule required for optimal results in anti-VEGF therapy. Currently, Iluvien is approved by the FDA for the treatment of DME.

### Next-generation anti-VEGF-A drugs

***Brolucizumab.*** Brolucizumab (Novartis Pharmaceuticals, Basel, Switzerland) is a humanized single-chain fragment variable binding to VEGF-A and interfering with activation of VEGF receptor 1 and 2 on endothelial cells. A 6-mg dose of brolucizumab delivers a molar dose which is about 11 and 22 times higher than aflibercept 2 mg and ranibizumab 0.5 mg, respectively. In addition, the low molecular weight and high concentration gradient between the vitreous and the retina may increase drug distribution to the target site of action, supporting effective control of anatomical disease activity. The drug has already been shown to be promising for the treatment of neovascularization associated with wet age-related macular degeneration in the HAWK and HARRIER clinical trials ^43^. Instead, in regard to DME, a phase 3, multi-center, double-masked clinical trial of this agent is ongoing to evaluate the efficacy and safety of brolucizumab in treatment of adult patients with visual disturbance due to DME in comparison with the administration of aflibercept ^44^. The results will be available in the next few years.

***Abicipar pegol.*** Abicipar pegol (Allergan Inc.) belongs to a novel class of small proteins that contain engineered ankyrin repeat domain(s) and bind to target proteins with high specificity and affinity. It is an antagonist of VEGF-A characterized by small size, high potency, and long intravitreal half-life. Results from the phase 2 study showed that abicipar pegol, injected every 8 or 12 weeks in patients affected by DME, offered the functional and anatomical effects with less frequent injections compared with ranibizumab over a 28-week period ^45^.

***Angiopoietin combination drugs.*** Together with VEGF-A, angiopoietin-2 (Ang-2) is considered a key factor in DME pathogenesis. Ang-2 is an antagonist of the Tie2 receptor tyrosine kinase on endothelial cells, counteracting vessel stabilization maintained through Ang-1–dependent Tie2 activation. The excess of Ang-2 and VEGF in the retinal tissues promotes vessel destabilization, vascular leakage, and neovascularization. Ang-2 is also involved in inflammatory pathways such as lymphocyte recruitment ^46– 49^. Different drugs targeting Ang-2, including RO6867461, a humanized full-length bispecific IgG1 antibody that selectively neutralizes VEGF-A and Ang-2, are in development. A phase III, double-masked, multi-center, randomized study is ongoing to evaluate the efficacy, safety, pharmacokinetics, and optimal treatment frequency of RO6867461 administered by intravitreal injection at 8-week intervals ^50^.

***Conbercept (KH902).*** Since its approval in China in 2013, conbercept has been used there for the treatment of neovascular age-related macular degeneration and other retinal vascular diseases, including DME ^51, 52^. However, it has not yet reached the market in other countries, although, given its excellent safety and efficacy profile, it has gained worldwide attention as a promising option. It is a recombinant fusion protein composed of the second IgG domain of VEGFR1 and the third and fourth domains of VEGFR2 to the constant region (Fc) of human IgG1. The structure is similar to that of aflibercept; however, conbercept has a VEGFR2 kinase insert domain receptor (KDR) Ig-like region 4 (KDRd4) that can improve the three-dimensional structure and increase dimer formation efficiency, thereby increasing the binding capacity of conbercept for VEGF. *In vitro* experiments have shown that binding of conbercept to VEGF is at least 30 times higher than that of ranibizumab and bevacizumab, achieving the same therapeutic effects but with a lower dose ^53– 55^.

In patients with DME, the FRONTIER ^56^ and SAILING ^57^ studies showed improvements in visual acuity and corresponding decreases in retinal thickness on optical coherence tomography. More trials on DME are in the planning stages.

### Combination drugs

***OPT-302.*** OPT-302 (Opthea Limited, South Yarra, Victoria, Australia) binds to VEGF receptor 2 and 3, neutralizing the activity of VEGF-C and -D. A combined use of OPT-302 with the currently available anti-VEGF-A therapy may be responsible for a true total VEGF inhibition. A phase 1b dose escalation study to evaluate OPT-302 in combination with aflibercept showed good results in vision improvement and reductions in retinal swelling ^58^. A phase 2a randomized controlled clinical study with OPT-302 is ongoing ^58^.

***ALG-1001.*** ALG-1001 (Allegro Ophthalmics, San Juan Capistrano, CA, USA) is a first-in-class integrin peptide therapy. The molecule is able to bind specific integrin receptor sites and works by affecting multiple angiogenic pathways and inflammation. ALG-1001 showed promising results in the phase IIb clinical trial that evaluated it as a sequential therapy or in combination with bevacizumab in patients with DME ^59, 60^.

### Suprachoroidal corticosteroid

Promising technologies for drug delivery currently under investigation include refillable surgical intravitreal implants, encapsulated cell technology, and suprachoroidal drug delivery ^61^. A phase 1 and 2 exploratory clinical trial examined suprachoroidal triamcinolone acetonide with and without intravitreal aflibercept, demonstrating increased efficacy and durability with the investigational drug ^62^. Used together with intravitreal aflibercept, suprachoroidal triamcinolone acetonide was generally well tolerated, and no treatment-related serious adverse events were reported in the TYBEE trial through the 24-week evaluation period ^63^.

## Management of macular edema: retinal vein occlusion

ME is the main cause of the deterioration of visual acuity in RVO ^64^. Actually, the first-line therapy in the treatment of ME secondary to RVO is represented by the intravitreal anti-VEGF injections of ranibizumab and aflibercept ^65, 66^. Moreover, intravitreal corticosteroid agents, such as triamcinolone and dexamethasone implant, have been evaluated ^67^ and are currently considered valid therapeutic options in RVO treatment because of their anti-inflammatory, anti-angiogenic, and anti-edema properties. They are able to improve visual acuity in the short term and have proven especially useful in pseudophakic patients or patients who do not experience significant intraocular pressure elevation with local steroid use. In regard to new therapeutic strategies, Iluvien is not yet approved by the FDA for ME secondary to RVO (see features of Iluvien in the “Iluvien” section above). Furthermore, two phase 3 studies are planned in order to evaluate the effect of brolucizumab in patients with ME secondary to RVO (see features of brolucizumab in the “Brolucizumab” section above) ^68, 69^.

## Management of macular edema: uveitis

ME is the most common cause of vision impairment in patients with uveitis, affecting 20 to 30% of them ^3, 70, 71^. It is typically associated with intermediate or posterior uveitis or panuveitis and is less frequent in anterior uveitis ^70, 72^. The treatment of uveitic macular edema (UME) can be challenging because of its relapsing nature and its tendency to persist in many cases despite good control of intraocular inflammation ^72^. Owing to a lack of prospective randomized controlled clinical trials, an internationally accepted approach to UME has yet to be established.

Below, we analyze the main treatments currently in use and the new therapeutic options for non-infectious UME. Infectious UME, conversely, is treated with targeted anti-microbial therapy, and anti-inflammatory treatments should be used after the active infection has been totally controlled ^73^.

### Corticosteroids

Corticosteroids are the mainstay of UME therapy because of their potent and fast-acting anti-inflammatory properties ^74^. However, short-term use is advised because of the harmful local (that is, cataract and intraocular pressure elevation) and systemic side effects ^75^.

***Topical.*** When inflammation in either subclinical or mild or UME is associated with anterior uveitis, a topical corticosteroid represents the first-line approach. The dose and duration of therapy depend on the underlying cause and the grade of inflammation, beginning with higher doses to be slowly tapered over time ^76^.

***Periocular.*** When inflammation is moderate to severe or UME is unresponsive to a topical approach, corticosteroids are administered through periocular depots. Triamcinolone acetonide and methylprednisolone acetate, administered via transeptal or sub-Tenon injections, are the most commonly employed ^77– 79^.

***Intravitreal.*** Triamcinolone acetonide (Taioftal ^®^, Sooft S.p.a., Montegiorgio, Italy) injection via pars plana has been largely used in UME, and the response has been excellent though brief. It appears to be more effective than periocular triamcinolone but with more side effects ^80^.

The fully biodegradable slow-release implant of dexamethasone (Ozurdex) is delivered for a period of 4 months and its safety and efficacy in non-infectious intermediate and posterior uveitis have been shown by the HURON study group and others ^81– 83^. Some studies reported the use of Ozurdex implant in persistent UME secondary to infectious uveitis after total control of active infection ^84, 85^.

Retisert (Bausch + Lomb, Rochester, NY, USA) is a non-biodegradable FAc 0.59-mg implant surgically implanted through the pars plana and sutured to the sclera. The MUST trial showed the overall superiority of FAc implant in controlling non-infectious uveitis compared with systemic therapy ^86^.

Iluvien is a non-biodegradable depot of 0.19-mg FAc approved for the treatment of DME. Iluvien is currently in randomized controlled clinical trials for the treatment of non-infectious uveitis ^87^.

***Systemic.*** Systemic corticosteroids are effective in treating UME, especially when bilateral, but systemic adverse effects prevent their long-term use. Prednisone is the most commonly used, and dosing and tapering should be individualized to the patient ^88^.

### Non-biologic immunomodulatory agents

Non-biologic immunomodulatory drugs are used as a second-line treatment for UME as corticosteroid-sparing agents. There are no randomized controlled trials toward the efficacy of these medications specifically on UME ^89^. Non-biologic immunomodulatory drugs include anti-metabolites (azathioprine, methotrexate, and mycophenolate mofetil), inhibitors of T-cell signaling (cyclosporine A, tacrolimus, and sirolimus), and alkylating agents (cyclophosphamide).

Azathioprine, methotrexate, and mycophenolate mofetil showed some efficacy in the treatment of UME secondary to different non-infectious uveitis ^90– 94^.

Cyclosporine A and tacrolimus are both effective in patients with uveitis, but few data exist when referring specifically to UME ^95, 96^. Intravitreal sirolimus presents a better safety profile than subcutaneous route with good control of inflammation and improvement of UME, but the results of SAVE studies demonstrated that frequent reinjections are needed ^97– 99^.

Cyclophosphamide inhibits both RNA transcription and DNA duplication. The SITE cohort study showed good control of inflammation with cyclophosphamide with a non-statistically significant increase in cancer-related mortality rates ^100^.

### Biologic immunomodulatory agents

Biologic immunomodulatory agents are humanized antibodies used for recalcitrant UME despite steroidal or traditional immunosuppressive treatments or both. Their main adverse effects include exacerbation of infectious and autoimmune diseases.

Tumor necrosis factor alpha blockers (anti-TNF-α) inhibit TNF-α, a pro-inflammatory cytokine considered crucial in the pathogenesis of different uveitis ^101^. Adalimumab (Humira; AbbVie Inc., North Chicago, IL, USA) is currently the only systemic non-corticosteroid agent which has been approved by the FDA for the treatment of non-infectious intermediate uveitis, posterior uveitis, or panuveitis in adults and children more than 2 years old. Many studies have shown improvement in macular thickness after systemic adalimumab but not after intravitreal administration ^101– 104^.

Infliximab (Remicade ^®^, Janssen Biotech, Inc., Horsham, PA, USA) is an anti-TNF-α administered intravenously. Like adalimumab, infliximab has shown positive results with systemic delivery and but not with an intravitreal route of administration ^105, 106^.

Golimumab (Simponi ^®^, Janssen Biotech Inc.) is another anti-TNF-α whose efficacy in UME has been demonstrated in case reports and small case series. Large control studies are required ^107, 108^.

Etanercept (Enbrel ^®^, Immunex Corporation, Seattle, WA, USA) is a fusion protein that blocks TNF-α and TNF-β and has been found to be ineffective for uveitis treatment. Its use has been discouraged in many case series ^93, 109^.

Tocilizumab (Actemra ^®^, Genentech Inc., San Francisco, CA, USA) blocks interleukin-6 (IL-6), a strong pro-inflammatory cytokine. Several studies have demonstrated efficacy in UME refractory to previous treatments in different types of uveitis ^110, 111^.

Rituximab (Rituxan ^®^, Genentech Inc.) targets CD20, which is the differentiation cluster of mature B cells. Multi-centered randomized controlled trials are lacking, but small case series have demonstrated beneficial effects in the treatment of non-infectious uveitis and UME ^112, 113^.

Many other molecules—such as abatacept (Orencia ^®^, Bristol-Myers Squibb Company, New York, NY, USA), a T-lymphocyte inhibitor; gevokizumab (XOMA 052, XOMA Corporation, Berkeley, CA, USA), an anti-IL-1β; ustekinumab (Stelara, Janssen-Cilag International NV, Beerse, Belgium), an anti-IL-12 and anti-IL-23; and filgotinib (GLPG0634), a janus kinase 1—are under investigation in different phase 2 clinical trials with promising results ^114– 120^.

## Conclusions

ME is a major complication of several vascular and inflammatory retinal diseases and multiple mechanisms are implicated in its development. During the acute phase of ME development, there is a visual function decline that often is reversible after the resolution of the edema (spontaneous resolution or after treatment). On the other hand, long-standing ME causes irreversible anatomical changes of the retina, leading to a permanent impairment of visual function. For these reasons, ophthalmologists should treat ME in the early phases of development in order to avoid irreversible damage of visual function.

The treatment of ME changes depending on the causative disease. In the DR, different intravitreal drugs for the treatment of DME, such as anti-VEGF injections (ranibizumab and aflibercept) and intravitreal corticosteroids (Ozurdex and Iluvien), are available. However, despite the strong efficacy achieved with these drugs, a significant proportion of patients with DME do not achieve anatomical or functional improvements. For this reason, new drugs are needed in the treatment of persistent or resistant forms of DME. Some of the drugs described here, especially brolucizumab and abicipar pegol, have been shown to be effective in preliminary studies, and these are probably the ones that have a better chance of soon being approved for the treatment of DME.

Also, for ME secondary to RVO, the therapeutic options are very similar to those for DME. However, Iluvien is not yet FDA-approved for the treatment of ME secondary to RVO.

ME secondary to uveitis is another important complication. In this case, a crucial role is played by corticosteroids (topical, periocular, intravitreal, and systemic) and non-biologic immunomodulatory drugs. However, in several cases, there is no resolution of UME, and the introduction into clinical practice of new biologic agents is an important new therapeutic option. Some of these new drugs (that is, adalimumab) have been approved by the FDA whereas several others are under investigation. Nonetheless, first it will be important to prove the safety and efficacy of these new drugs. Only after that will we have new therapeutic options in the treatment of UME.

## Abbreviations

Ang, angiopoietin; DME, diabetic macular edema; DR, diabetic retinopathy; FAc, fluocinolone acetonide; FDA, US Food and Drug Administration; IL, interleukin; ME, macular edema; RVO, retinal vein occlusion; TNF, tumor necrosis factor; UME, uveitic macular edema; VEGF, vascular endothelial growth factor
